# Two Rare Cases of Tinea Corporis Caused by Trichophyton verrucosum and Trichophyton interdigitale in a Teenage Girl

**DOI:** 10.7759/cureus.5325

**Published:** 2019-08-05

**Authors:** Caroline B Crain, Arathi Rana, Frank T Winsett, Michael G Wilkerson

**Affiliations:** 1 Dermatology, University of Texas Medical Branch, Galveston, USA

**Keywords:** tinea corporis, trichophyton verrucosum, trichophyton interdigitale, zoophilic dermatophytes, majocchi's granuloma

## Abstract

We present two cases of tinea corporis caused by *Trichophyton verrucosum* and *Trichophyton interdigitale* in a teenage girl who works with farm animals. We describe the differences in presentation between zoophilic dermatophytes and anthropophilic dermatophytes. Also, we report some of the typical features of the two rare species, *T. verrucosum* and *T. interdigitale*. This case is significant to dermatology as it raises awareness about these uncommon zoophilic dermatophytoses and demonstrates the importance of educating patients about their mode of infection.

## Introduction

Dermatophytoses, also known as tinea or ringworm, are contagious mycoses of the skin that can cause infection in a wide range of mammals including pet animals and livestock. The three most common zoonotic dermatophytes are *Microsporum canis* primarily from cats and dogs, *Trichophyton mentagrophytes* primarily from rodents, and *Trichophyton verrucosum *primarily from cattle and other ruminants [1]. Herein, we present two cases of tinea corporis caused by *Trichophyton verrucosum* and *Trichophyton interdigitale *in a teenage girl who works with farm animals.

## Case presentation

**Figure 1:**
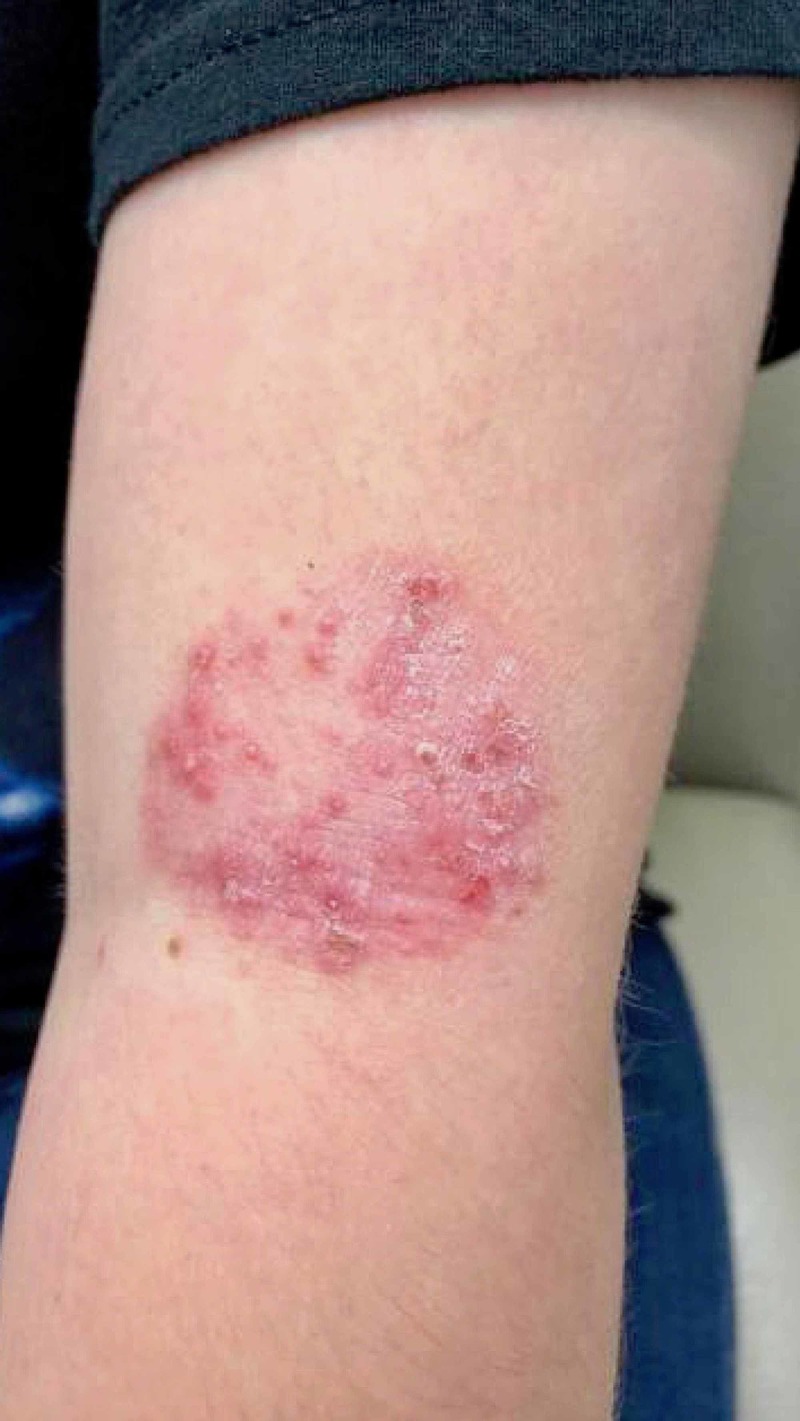
Patient's initial presentation revealing annular, erythematous, indurated plaque with pustules and excoriations present on the patient's left arm consistent with tinea corporis due to T. verrucosum.

**Figure 2:**
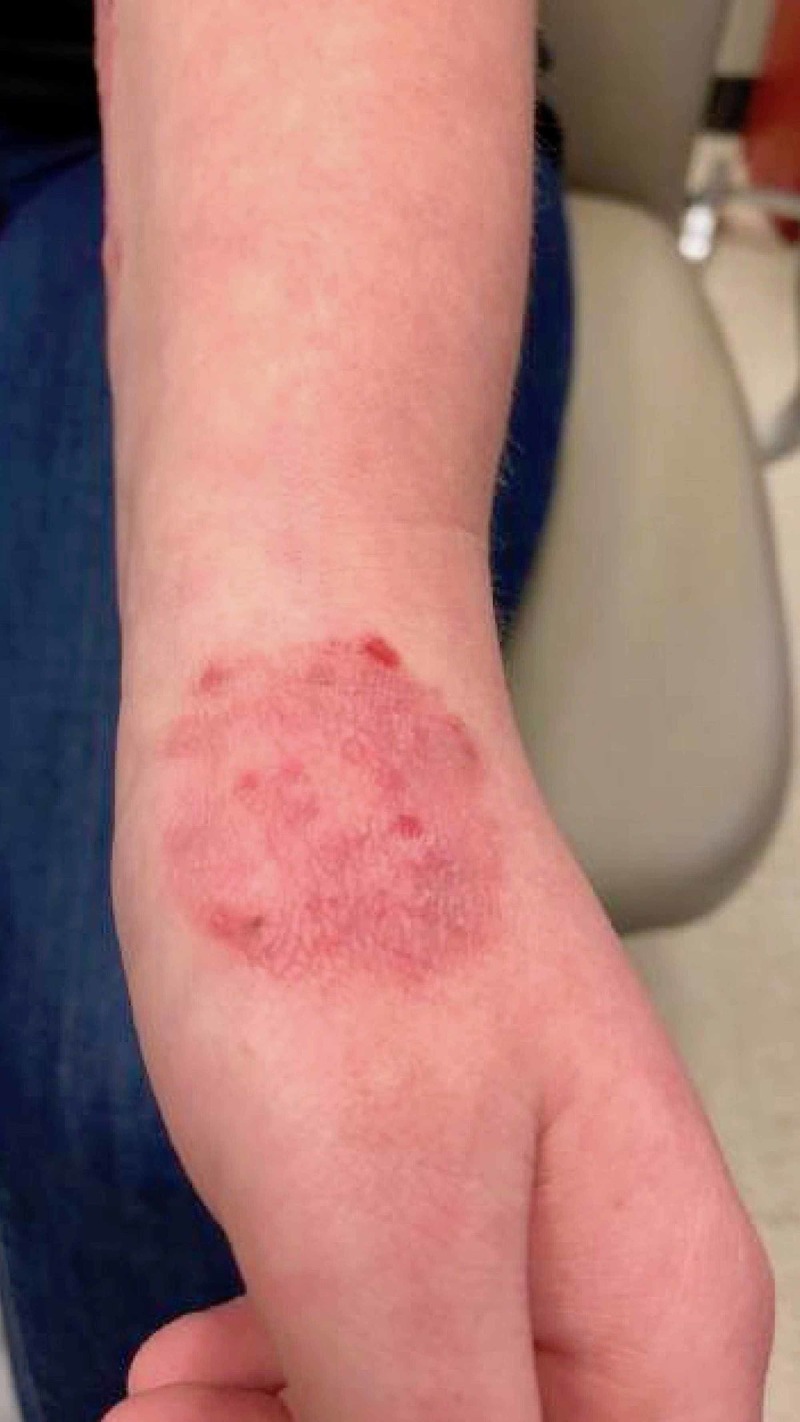
Patient's initial presentation revealing annular, erythematous, indurated plaque present on the patient's left hand consistent with tinea corporis due to T. verrucosum.

A 17-year-old female presented to the dermatology clinic with a pruritic rash on her left arm that had been present for two months. The patient was an aspiring veterinarian and worked with goats, lambs, and sheep in her spare time. Physical examination revealed annular erythematous indurated plaques with several pustules and excoriations on her left shoulder, arm, and hand (Figures 1, 2).

The patient had a negative potassium hydroxide (KOH) preparation in clinic, but fungal culture and internal transcribed spacer (ITS) sequencing were consistent with *Trichophyton verrucosum*. A punch biopsy revealed prominent acute and chronic perifollicular inflammation consistent with a diagnosis of Majocchi’s granuloma. After one month of treatment with terbinafine 250 mg, the lesions resolved.

About a year later, the patient presented again with a rash on her arms and left leg that had been present for several weeks. She reported continued exposure to farm animals. Physical examination revealed scattered erythematous papules on her bilateral arms, left leg, and chest. Tinea corporis was suspected, but a KOH preparation was negative in clinic. However, a fungal culture confirmed the diagnosis and was positive for *Trichophyton interdigitale*. It was successfully treated with another course of terbinafine.

## Discussion

Tinea corporis usually presents as a pruritic, erythematous, scaling papule or plaque that spreads centrifugally resulting in an annular lesion [1]. Zoophilic dermatophytes tend to cause significant inflammation and can lead to the development of crusts, vesicles, papules, or pustules [2]. Majocchi’s granuloma develops when fungal organisms invade the dermis creating granulomatous inflammation around the hair follicle [3]. Typically, fungal hyphae and spores can be detected within and around the follicle [3].

*T. verrucosum*, a zoophilic dermatophyte species, is the most common cause of ringworm in cattle and other ruminants [1]. It is more prevalent in younger animals and is transmitted by direct contact with affected animals or through contaminated fomites [4]. *T. interdigitale* is one of four species grouped under the *T. mentagrophytes* complex and includes anthropophilic and zoophilic strains [5]. The zoophilic strains typically cause more severe inflammatory lesions than the anthropophilic strains, and patients usually have a history of animal contact [6]. Although it may not alter the treatment plan, strain differentiation is important for determining the infection source and for epidemiological surveillance. ITS sequencing of ribosomal deoxyribonucleic acid (DNA) is considered the gold standard for molecular identification of dermatophytes [7].

## Conclusions

In conclusion, this case raises awareness about the two zoophilic dermatophytes, *T. verrucosum* and *T. interdigitale *and demonstrates the importance of educating patients about their mode of infection. ITS sequencing should be considered in patients with exposure to farm animals who are diagnosed with tinea corporis.
